# Supplementary material for: Interest in public involvement, engagement and participation in research among patients with asthma: A secondary analysis of the EPI-ASTHMA study
Source: PLoS One. 2025 Sep 12;20(9):e0332220. doi: 10.1371/journal.pone.0332220 (PMC12431402; doi:10.1371/journal.pone.0332220)
Supplement: Attachment — Submitted filename: Response_Letter PLOS ONE.docx [file pone.0332220.s003.docx]

Dear Prof. Manuela Mendonça Figueirêdo Coelho,
Academic Editor of PLOS One,

April 28th, 2025

Title: Interest in Public Involvement, Engagement and Participation in Research among Patients with Asthma: A Secondary Analysis of the EPI-ASTHMA Study

Manuscript ID: PONE-D-25-04695

Dear Prof. Manuela Mendonça Figueirêdo Coelho,

Thank you for considering our manuscript. Below you will find a point-by-point reply to the issues raised. We would like to thank you for the useful comments. All changes made in the manuscript are highlighted.

I am looking forward to hearing from you.

Yours sincerely,

Cristina Jácome

**Academic Editor**

As an optional suggestion, aiming only to further strengthen the final submission, I would like to propose

a few minor improvements:

1. Multicollinearity assessment: Although the absence of multicollinearity evaluation was acknowledged in the response letter, it would be advisable to briefly mention this in the manuscript’s Limitations section. A sentence such as the following could be added:

“Another limitation is that multicollinearity between predictors was not assessed, which could affect the interpretation of regression results.”

Response: Discussion of this limitation has been added. Please see lines 261-263.

2. Consistency of data collection dates: There is a minor inconsistency regarding the final date of data collection (December 2023 vs. February 2024). I recommend verifying and ensuring consistency throughout the manuscript.

Response: Data collection closed in March 2024. This has now been clarified. Please see line 101.

3.Title formatting: Following PLOS ONE formatting style, please consider capitalizing the main words in the title as follows:

Interest in Public Involvement, Engagement and Participation in Research among Patients with Asthma: A Secondary Analysis of the EPI-ASTHMA Study.

Response: Title has been edited as suggested.
