# Supplementary material for: Interest in public involvement, engagement and participation in research among patients with asthma: A secondary analysis of the EPI-ASTHMA study
Source: PLoS One. 2025 Sep 12;20(9):e0332220. doi: 10.1371/journal.pone.0332220 (PMC12431402; doi:10.1371/journal.pone.0332220)
Supplement: Attachment — Submitted filename: Response to Reviewers.docx [file pone.0332220.s004.docx]

Dear Prof. Manuela Mendonça Figueirêdo Coelho,
Academic Editor of PLOS One,

May 23th, 2025

Title: Interest in Public Involvement, Engagement and Participation in Research among Patients with Asthma: A Secondary Analysis of the EPI-ASTHMA Study

Manuscript ID: PONE-D-25-04695

Dear Prof. Manuela Mendonça Figueirêdo Coelho,

Thank you for considering our manuscript. Below you will find a point-by-point reply to the issues raised. We would like to thank you for the useful comments. All changes made in the manuscript are highlighted in blue.

I am looking forward to hearing from you.

Yours sincerely,

Cristina Jácome

**Third revision**

**Academic Editor**

If you have made the corrections requested by the reviewers below, please send a new file with the corrections marked in blue, as well as a response letter regarding each correction.

Response: Something was probably lost in the process. In all interactions (R1 and R2) I have uploaded a point-by-point response letter, together with a manuscript version with changes highlighted. In this response letter, you can find all responses provided in these two interactions. Please see below. In addition, the manuscript is now provided with all corrections from these two interactions marked in blue.

**Second revision**

**Academic Editor**

As an optional suggestion, aiming only to further strengthen the final submission, I would like to propose

a few minor improvements:

1. Multicollinearity assessment: Although the absence of multicollinearity evaluation was acknowledged in the response letter, it would be advisable to briefly mention this in the manuscript’s Limitations section. A sentence such as the following could be added:

“Another limitation is that multicollinearity between predictors was not assessed, which could affect the interpretation of regression results.”

Response: Discussion of this limitation has been added. Please see lines 261-263.

2. Consistency of data collection dates: There is a minor inconsistency regarding the final date of data collection (December 2023 vs. February 2024). I recommend verifying and ensuring consistency throughout the manuscript.

Response: Data collection closed in March 2024. This has now been clarified. Please see line 101.

3.Title formatting: Following PLOS ONE formatting style, please consider capitalizing the main words in the title as follows:

Interest in Public Involvement, Engagement and Participation in Research among Patients with Asthma: A Secondary Analysis of the EPI-ASTHMA Study.

Response: Title has been edited as suggested.

**First revision March 26th**

**Academic Editor**

When submitting your revision, we need you to address these additional requirements.

1. Please ensure that your manuscript meets PLOS ONE's style requirements, including those for file naming. The PLOS ONE style templates can be found at

<https://journals.plos.org/plosone/s/file?id=wjVg/PLOSOne_formatting_sample_main_body.pdf>

<https://journals.plos.org/plosone/s/file?id=ba62/PLOSOne_formatting_sample_title_authors_affiliations.pdf>

Response: The manuscript has been modified to meet PLOS ONE style, namely, abstract headings were removed, manuscript heading styles edited; title page edited.

2. Thank you for stating the following in the Competing Interests section:

“Filipa Bernardo (FB) is an employee of AstraZeneca, Produtos Farmacêuticos SA. The remaining authors have no conflicts of interest to declare.”

We note that one or more of the authors are employed by a commercial company: AstraZeneca

a. Please provide an amended Funding Statement declaring this commercial affiliation, as well as a statement regarding the Role of Funders in your study. If the funding organization did not play a role in the study design, data collection and analysis, decision to publish, or preparation of the manuscript and only provided financial support in the form of authors' salaries and/or research materials, please review your statements relating to the author contributions, and ensure you have specifically and accurately indicated the role(s) that these authors had in your study. You can update author roles in the Author Contributions section of the online submission form.

Please also include the following statement within your amended Funding Statement. “The funder provided support in the form of salaries for authors [insert relevant initials], but did not have any additional role in the study design, data collection and analysis, decision to publish, or preparation of the manuscript. The specific roles of these authors are articulated in the ‘author contributions’ section.” If your commercial affiliation did play a role in your study, please state and explain this role within your updated Funding Statement.

b. Please also provide an updated Competing Interests Statement declaring this commercial affiliation along with any other relevant declarations relating to employment, consultancy, patents, products in development, or marketed products, etc.

Within your Competing Interests Statement, please confirm that this commercial affiliation does not alter your adherence to all PLOS ONE policies on sharing data and materials by including the following statement: "This does not alter our adherence to PLOS ONE policies on sharing data and materials.” (as detailed online in our guide for authors http://journals.plos.org/plosone/s/competing-interests) . If this adherence statement is not accurate and there are restrictions on sharing of data and/or materials, please state these. Please note that we cannot proceed with consideration of your article until this information has been declared.

Please include both an updated Funding Statement and Competing Interests Statement in your cover letter. We will change the online submission form on your behalf.

Response: The Funding Statement and Competing Interests Statement were updated and added in the Cover Letter as requested. Please see below:

Funding: This study was sponsored and funded by AstraZeneca. The funder provided support in the form of salaries for authors FB, but did not have any additional role in the study design, data collection and analysis, decision to publish, or preparation of the manuscript.

Conflict of interest: We have read the journal’s policy and the authors of this manuscript have the following competing interests: FB is an employee of AstraZeneca, Produtos Farmacêuticos SA. FB does not have any other conflicts of interest to declare. This does not alter our adherence to PLOS ONE policies on sharing data and materials. The remaining authors have no conflicts of interest to declare.

3. We note that you have indicated that there are restrictions to data sharing for this study. For studies involving human research participant data or other sensitive data, we encourage authors to share de-identified or anonymized data. However, when data cannot be publicly shared for ethical reasons, we allow authors to make their data sets available upon request. For information on unacceptable data access restrictions, please see <http://journals.plos.org/plosone/s/data-availability#loc-unacceptable-data-access-restrictions>

Before we proceed with your manuscript, please address the following prompts:

a) If there are ethical or legal restrictions on sharing a de-identified data set, please explain them in detail (e.g., data contain potentially identifying or sensitive patient information, data are owned by a third-party organization, etc.) and who has imposed them (e.g., a Research Ethics Committee or Institutional Review Board, etc.). Please also provide contact information for a data access committee, ethics committee, or other institutional body to which data requests may be sent.

b) If there are no restrictions, please upload the minimal anonymized data set necessary to replicate your study findings to a stable, public repository and provide us with the relevant URLs, DOIs, or accession numbers. Please see <http://www.bmj.com/content/340/bmj.c181.long> for guidelines on how to de-identify and prepare clinical data for publication. For a list of recommended repositories, please see https://journals.plos.org/plosone/s/recommended-repositories. You also have the option of uploading the data as Supporting Information files, but we would recommend depositing data directly to a data repository if possible.

Please update your Data Availability statement in the submission form accordingly.

Response: The Data Availability statement has been updated: “There are ethical or legal restrictions on sharing a de-identified data set, because that the data contain potentially identifying or sensitive patient information and the ethics committee imposed them. This study was approved by the ethics committees of the Regional Health Administrations of North (CE/2022/117), Center (27/2021), Lisbon and Tagus Valley (2775/CES/2022), Alentejo (11/CE/2022) and Algarve (1/2022) and of the Local Health Units of Matosinhos (38/CES/JAS) and Alto Minho (38/2021). The original approval from the Ethical Review Boards did not include direct and free data access and therefore, data cannot be made freely available. All data supporting the findings are available from the Faculty of Medicine of University of Porto for all interested researchers who meet the criteria for access to confidential data. Please contact Cristina Jácome, the corresponding author at cjacome@med.up.pt, or the Faculty of Medicine of University of Porto at fmup@med.up.pt to request the data.

I would also like to stress that a similar Data Availability Statement was published in a previous secondary analysis also published in PLOS One (https://journals.plos.org/plosone/article?id=10.1371/journal.pone.0317614).

**Reviewer #1**

Cunha and colleagues sought to identify the factors associated with the interest in being involved in a Patient and Public Involvement Engagement and Participation (PPIEP) among patients with asthma. It is a secondary analysis of the EPI-ASTHMA nationwide asthma prevalence study where a total of 502 participants with asthma (52 ±15.9 years, 64.1% females) from 38 primary healthcare centers from mainland Portugal were analyzed. They found that a total of 218 (43.4%) participants with asthma were interested in being involved in the Patient and Public Involvement Engagement and Participation (PPIEP). They concluded that two out of five patients with asthma were interested in participating in PPIEP initiatives. They also indicated that by understanding patient characteristics willing to participate in PPIEP, they could develop strategies to reach a wider audience and make PPIEP more inclusive. The identified associated factors that they observed need to be further investigated. This is an interesting report based on an effort to analyze willingness of patients to participate in asthma research. I have a few suggestions to improve the presentation along with a few minor comments for corrections to the text:

1. Title – if necessary, you could eliminate the Patient and Public Involvement Engagement and Participation portion.

Response: The title has been reduced, but the expression “Patient and Public Involvement, Engagement, and Participation,” as defined by the UK National Institute for Health and Care Research, has been retained. This term was recently defined to replace previous PPI and PPIE and unfortunately it is still relatively unfamiliar to the general scientific community, and ongoing efforts are required to enhance its recognition and understanding. Please see lines 1-2: “Interest in public involvement, engagement and participation in research among patients with asthma: A secondary analysis of the EPI-ASTHMA study”

2. Abstract, p. 2, line 54 – do you have specific strategies in mind that could be included?

Response: While we have included examples of specific strategies in the Discussion section (lines 237-246), we believe it would not be appropriate to add them to the abstract's conclusion. These suggestions are preliminary and were not explicitly discussed or validated by the study participants, so presenting them in the abstract could overstate their significance. We are happy to clarify further or discuss alternative adjustments if needed.

3. Introduction – p. 3 – line 72 - was the Patient and Public Involvement Engagement and Participation initiative set up for one study or for many studies? Perhaps a little more background on the development of this program would be helpful. How long has it been going on? Has it been successful? Will it continue?

Response: In lines 72-73 we are defining PPIE in a broad way, regardless of the chronic condition using the most recent definition of the UK National Institute for Health and Care Research (NIHR).In the subsequent lines (74-80), we are trying to show to the reader that recent evidence from original and synthesis studies(ref 10, 11 and 12) demonstrate that PPIE in health research has important advantages. From lines 80-83, we point out the current challenges in implementing PPIE.

All this evidence came from different studies and not from a single experience or program. We tried to make that clear. Please see lines 66-73: “A review of reviews showed that incorporating PPIEP into health research and healthcare can be a powerful tool for identifying patient needs and optimising treatment approaches [11]. PPIEP aims to improve patient outcomes and reduce healthcare costs by empowering patients to make informed decisions and promoting open discussions about disease symptoms and comorbidities [12]. Yet, previous experiences in other context or conditions demonstrate that the effectiveness of PPIEP depends on various factors, including the quality of the relationship between researchers, patients, and the public [13], accessibility of information and training opportunities [14], as well as the health limitations and sociodemographic characteristics of the participants [15].”

4. Introduction, p. 4, lines 104 to 105 – what is the goal of this program after having collected this information? In what manner will it continue?

Response: The current paper does not address the next steps. The current paper focuses on raising awareness that patients are interested in PPIEP initiatives related to asthma. But it also points out that we are captivating only patients with certain characteristics. If broader patient involvement is desired in the future, alternative strategies will be needed to reach and engage a more diverse group.

5. Study Design, p.4 – a little more information about the Patient and Public Involvement Engagement and Participation program would be helpful in terms of the scope of the program, target age, entry criteria, exclusion criteria and also the anticipated burden of the study PPIEP is recruiting for and whether that was included in the invitation to particpate. What were potential participants told would be done with their data and the reason for providing their permission?

Response: Thank you for your valuable comment. We would like to clarify that at the time of this study, no formal PPIEP program existed. The research team conducted this work specifically to assess the potential feasibility of establishing such a network in future. We have now included additional details about the specific question posed to participants to assess their interest. Please see page lines 129-133: “At the end of the stage 2 visit, patients answered a question regarding PPIEP interest (‘We intend to constitute a group of people with asthma with a more active role in research. Your participation will be occasional and will contribute to enrich the research projects - from their conception to the final result - with the perspective and needs of people who deal with asthma day by day. Are you interested in collaborating with this group?’).”.

6. Participants, p.4 – What was the target population surveyed? Were pediatric patients purposely excluded? Was there data collected on their prior participation in asthma or any other studies?

Response: Thank you for your comment. We would like to clarify that the assessment of PPIEP interest was conducted among all adult patients with asthma during stage 2 of the Epi-asthma study. As this is a secondary analysis of Epi-asthma—a study primarily designed to assess adult asthma prevalence in Portugal—our findings are inherently limited to the adult population. Pediatric patients were not purposely excluded, yet we acknowledge this as a limitation of our research, which we have now explicitly addressed in our discussion section. Please see lines 251-255: “As this was a secondary analysis, our study was limited to the available sample from the main study, which included only adult participants. We recognize this as an important limitation and agree that future research investigating PPIEP interest and implementation strategies should actively include paediatric populations and their caregivers to ensure more comprehensive representation.”

7. Discussion, p. 10, line 216 – what is the description of the disadvantaged population in Portugal? Is it low-income families, homeless, a particular race/ethnicity?

Response: We have now clarified this. Please see lines 210-212: “But we need to consider existing disparities in the adoption and utilization of these technologies, often leaving disadvantaged groups, particularly individuals with low education levels; low incomes, immigrants, further behind [34].”

8. Discussion, p. 10, line 242 – What would you consider an important level of diversity and representative population for an asthma study in Portugal? Same is true for p. 11, line 244, what is the underrepresented groups in Portugal? That could be different from other countries, such as the United States.

Response: We have clarified above what are the common underrepresented groups in Portugal. In this paragraph we have tried to provide more information on the known barriers to participation by these groups and also highlighted the need to gather evidence of specific inclusion strategies. Please see lines 237-246: “While our study provides valuable insights into the factors associated with patient interest in PPIEP, it also underscores the need to address potential barriers to participation, such as limited accessibility of engagement formats (e.g., rigid meeting times); lack of compensation for participation; unfamiliarity with research processes.[24, 37] Ensuring diverse and representative involvement in PPIEP is crucial for enhancing the translation of research into practice and promoting equitable healthcare. Future research should focus on identifying barriers faced by underrepresented groups and on the effectiveness of targeted inclusion strategies to foster a more inclusive approach to PPIEP. Approaches warranting investigation include community-partnered recruitment through trusted local organizations, development of plain-language materials, and implementation of flexible participation modalities (both virtual and in-person).[38]”

9. Discussion, p. 11, line 249 – What is secondary care patients?

Response: We were referring to secondary care setting, but the sentence has now been rewritten to improve clarity. Please see lines 249-251: “However, our primary care-based recruitment strategy may have limited our ability to capture perspectives from patients with severe asthma, as these individuals are more likely to be managed in secondary care settings.”

10. Discussion, p. 11 – it might be of interest to compare your population findings to published studies on the various levels of asthma severity.

Response: Unfortunately there are no studies with more severe patients with asthma. The similar works we found are all cited in the current manuscript. That is why we discuss that in the limitations section and advise for further research. Please lines 249-251: “However, our primary care-based recruitment strategy may have limited our ability to capture perspectives from patients with severe asthma, as these individuals are more likely to be managed in secondary care settings.”

11. Discussion, p.11, line 260 – What is your future vision for the application of this data and meeting your goals for studies? Will PPiEP continue its work and for what purpose?

Response: We answered these two points together. We believe that the paragraph (lines 237-246), which has been substantially enriched to clarify our vision on PPIEP and needed strategies. PPIEP will improve research but if done properly and involving all regardless of their characteristics: “While our study provides valuable insights into the factors associated with patient interest in PPIEP, it also underscores the need to address potential barriers to participation, such as limited accessibility of engagement formats (e.g., rigid meeting times); lack of compensation for participation; unfamiliarity with research processes.(24, 37) Ensuring diverse and representative involvement in PPIEP is crucial for enhancing the translation of research into practice and promoting equitable healthcare. Future research should focus on identifying barriers faced by underrepresented groups and on the effectiveness of targeted inclusion strategies to foster a more inclusive approach to PPIEP. Approaches warranting investigation include community-partnered recruitment through trusted local organizations, development of plain-language materials, and implementation of flexible participation modalities (both virtual and in-person).(38)”

12. Discussion, Conclusions, p. 11, line 266 – What strategies do you propose?

Response: We answered these two points together. We believe that the paragraph (lines 237-246), which has been substantially enriched to clarify our vision on PPIEP and needed strategies. PPIEP will improve research but if done properly and involving all regardless of their characteristics: “While our study provides valuable insights into the factors associated with patient interest in PPIEP, it also underscores the need to address potential barriers to participation, such as limited accessibility of engagement formats (e.g., rigid meeting times); lack of compensation for participation; unfamiliarity with research processes.(24, 37) Ensuring diverse and representative involvement in PPIEP is crucial for enhancing the translation of research into practice and promoting equitable healthcare. Future research should focus on identifying barriers faced by underrepresented groups and on the effectiveness of targeted inclusion strategies to foster a more inclusive approach to PPIEP. Approaches warranting investigation include community-partnered recruitment through trusted local organizations, development of plain-language materials, and implementation of flexible participation modalities (both virtual and in-person).(38)”

13. Discussion, Conclusions, p. 11, line 267 – How do you define a diverse perspective for your purposes in conducting asthma studies?

Response: We believe this has been clarified in the previous commnent.

14. References – Check to see that your author format for references is consistent with journal requirements.

Response: References have now been updated to follow PLOS ONE requirements. Please see References list.

Minor corrections:

1. Page 9, line 201- ….did not address…

Response: This has been modified. Please see page, line 195: “that also could impact patient interest and were not addressed in our study”

2. Page 10, line 215 - ….to be cautious regarding…

Response: This has been modified. Please see page, line 210: “But we need to consider existing”

**Reviewer #2**

Your study is interesting and beneficial to those interested in patient recruitment. I think it should be published. Your enrollment and demographics are quite similar to what our group found when we investigated participation in survey-based research during the COVID pandemic in the US. Because the EPI-ASTHMA study is still in press, I am unable to find all of the details related to recruitment (which is why I said details were not available).

Response: The Epi-asthma study is now online, you can find it here: https://pubmed.ncbi.nlm.nih.gov/40094487/. Sorry for the inconvenience.

For the most part, the research and conclusions are sound. However, one obvious source of bias is that you recruited participants by phone. I think the reason smartphone ownership was so strongly tied to participation was because of the recruitment method. The recruitment method could also introduce bias for other factors such as age, gender, degree of asthma control, etc. However, smartphone usage is the most obvious. This bias definitely needs to be mentioned in your discussion. And in fact, one could argue that smartphone ownership should not be included in your analysis at all.

Response: Regarding the recruitment, first adult individuals were randomized from the lists of the 38 primary care centres distributed across Portugal mainland. Then the invitation was made by phone using the telephone numbers provided in the primary care electronic health record (HER). Contact numbers were not restricted to mobile phones as a high percentage of participants only had in their EHR fixed/land-line numbers. But even though, when the patient was not reached in the primary contact number, the second available number (from a caregiver or person of contact) was used. For this reason, we do not believe that telephone recruitment constituted a study bias nor removes the interest of investigating the ownership of smart devices. In fact, in a previous study of our team, in which patients were recruited in secondary care settings, a similar number of smart device ownership was also observed (76% in the present study and 75% in the previous one, https://www.jiaci.org/revistas/vol30issue2_7-3.pdf).

I noticed that the number of participants in the study was much less than expected. Without access to the EPI-ASTHMA manuscript I do not know why, but this may be worth mentioning. There was also no explanation as to why 16 participants were excluded from your analysis.

Response: The Epi-asthma study achieved the number of participants anticipated. From the 22 075 invited, 30% declined the invitation (n=6786), which is perfectly aligned with real-world observational studies. Regarding this secondary analysis, the 16 individuals did not answer the PPIEP question and were not included. This has now been clarified. Please see lines 152-154: “A total of 518 participants were diagnosed with asthma in the Epi-asthma study but 16 did not answered the PPIEP question. Therefore, 502 (97%, mean age 52 ± 15.9 years) were included in this secondary analysis.”

There were grammar/syntax errors in lines 134, 201, and 202. The sentence starting on line 215 does not make sense and needs to be reworked.

Response: These sentences have been rewritten to avoid errors.

Please see lines 125-126: “CARAT is a self-reported questionnaire with a total score (CARAT-T) ranging between 0–30 points.”

Please see lines 196-198:” The researcher-participant relationship is also an important factor to consider in PPIEP [28]. Positive engagement dynamics and trust-building measures are essential for sustaining participant motivation and continued involvement throughout the research process [24].”

Please see lines 210-212:” But we need to consider existing disparities in the adoption and utilization of these technologies, often leaving disadvantaged groups, particularly individuals with low education levels; low incomes, immigrants, further behind [34].”

**Reviewer #3**

Dear Authors, Thank you for your efforts. There are some major points that need improvement:

Title: The title would benefit from reducing the words. Some words like “factors” are vague and better not to be used in title.

Response: The tile has now been reduced and word ‘factors’ removed. Please see lines 1-2: “Interest in public involvement, engagement and participation in research among patients with asthma: A secondary analysis of the EPI-ASTHMA study”.

Abstract:

Line 45: “total of 502 participants with asthma (52 ± 15.9 years, 64.1% females)”: Usually these data are described in result not method.

Response: These data has moved to the Results and Methods section was completed with the setting and population.

Methods, Please see lines 31-33: “This is a secondary analysis of the EPI-ASTHMA nationwide asthma prevalence study with adult patients with asthma recruited from 38 primary healthcare centres from mainland Portugal.”

Results, Please see lines 35-36: “A total of 502 participants with asthma (52 ± 15.9 years, 64.1% females) were analysed”

Line 47: A multivariable logistic regression model was built: Please identify what variable the model was built for.

Response: This has now been clarified in abstract. Please see lines 34-35: “A multivariable logistic regression model was built to explore associations between participants’ characteristics and PPIEP interest.”

Introduction: The research aim is clear but brief. Consider adding 1-2 sentences about the significance of identifying these factors and potential implications.

Response: Thank you for your suggestion. A sentence about the possible implications has been added. Please see line 94-96: “Understanding these factors is critical for designing equitable engagement strategies and can guide researchers and policymakers in creating inclusive PPIEP frameworks.”

Method:

- The method section is a bit unclear. It is not clearly mentioned if the study is a secondary analysis of data available, or they have actively gathered data. For example, they have stated that they have gotten consent from the participants, meaning active participation. However, in all details they are talking about a third party data gathering refereeing other articles.

Response: Thank you for your comment. We would like to clarify that the assessment of PPIEP interest was conducted among all adult patients with asthma during stage 2 of the Epi-asthma study and specific data collection was conducted. The research team conducted this work specifically to assess the potential feasibility of establishing a PPIEP network in future.

- The key variable about PPIEP interest is not well described. Add details about how this was measured.

Response: We have now included additional details about the specific question posed to participants to assess their interest. Please see lines 129-133: “At the end of the stage 2 visit, patients answered a question regarding PPIEP interest (‘We intend to constitute a group of people with asthma with a more active role in research. Your participation will be occasional and will contribute to enrich the research projects - from their conception to the final result - with the perspective and needs of people who deal with asthma day by day. Are you interested in collaborating with this group?’).”.

- Consider adding information about how you handled missing data

Response: The 16 individuals with missing data were excluded from the analysis. This has been clarified. Please see lines 152-153: “A total of 518 participants were diagnosed with asthma in the Epi-asthma study but 16 did not answered the PPIEP question. Therefore, 502 (97%, mean age 52 ± 15.9 years) were included in this secondary analysis.”

In table 1, the missing values for each of the variables used to describe the sample are acknowledged. No specific procedure was done to deal with missing data.

- Specify which variables were tested in the simple logistic regressions

Response: Variables have now been specified. Please see lines 140-143: “Simple logistic regressions were used to explore associations between participants’ characteristics (age, sex, marital status, education, occupation, geographic region, body mass index, smoking habits, smartphone ownership, use of health and fitness apps, number of comorbidities, exacerbations in the past year, unscheduled appointments with specialist in the past year, CARAT, mini-AQLQ ) and PPIEP interest.”

- Consider mentioning how you assessed for multicollinearity in your regression models

Response: While we recognize that multicollinearity assessment is an important step in regression analysis, this evaluation was not conducted in our study. We acknowledge this as a limitation that should be addressed in future research with larger samples to ensure robust interpretation of associated factors.

Result:

- Line 166-171: When reporting the multivariable results, consider adding p-values alongside the odds ratios and confidence intervals.

Response: It was an author’s choice to report confidence intervals without accompanying p-values. Presenting both measures would create statistical redundancy. In addition, confidence intervals provide more complete information about effect size precision. We believe this approach aligns with current methodological recommendations emphasizing estimation over binary hypothesis testing. However, we appreciate the reviewer's perspective and remain open to reconsidering this presentation in future revisions if deemed necessary.

- The interpretation of OR 1.53 (95%CI 1.00-2.33) for females should be approached with caution as the lower bound of the confidence interval is exactly 1.00, suggesting borderline significance.

Response: Thank you for your comment. We have edited both results and discussion section to highlight this fact.

Results, please see lines 167-168: “Being female demonstrated a borderline significant association (adjusted OR 1.53, 95%CI 1.00-2.33).”

Discussion, please lines 220-221: “Although borderline significant, being a female demonstrated an association with higher odds of being interested in PPIEP.”

- There is a problem in Smartphone ownership data percentage. 198 out of 218 would be 90.8%, not 93.6%. Please check all data again.

Response: This has now been corrected and all data checked for accuracy. Please see table 1.

Discussion

Try organizing paragraphs more thematically.

Response: We have reorganized the discussion section, highlighting first the high frequency of interest and then the discussion of factors from the most to the least significant. The discussion of the strengths and limitations is provided at the end of the section and has also been enriched.

Some minor editions:

1. In line 63, “This variation” could be clearer by specifying “This variation in asthma control”.

Response: We have accepted your suggestion. Please lines 50-51: “This variation in asthma control is influenced by several factors, including adherence to treatment, inhaler technique, comorbid conditions, and environmental factors such as exposure to allergens or smoke and respiratory tract infections [4, 5].”

2. I am not sure whether PCC stands for primary healthcare centres. (Line 110)

Response: We have removed that abbreviation. Please see lines 100-101: “We used data collected between May 2021 and December 2023 in 38 primary healthcare centres from mainland Portugal.”

3. Line 110: “Data was accessed” should be “Data were accessed”.

Response: We did not identify the expression “Data was accessed” in our manuscript.

4. More clearly define “current asthma” versus “possible asthma” or reference where these definitions can be found.

Response: Reference 2 is now available and explains clearly the difference between the two definitions. We believe it is not worth to clarify this in the current work as an open access reference is available if the reader wants to obtain further information.

5. Line 192: “more women than man” shou

Response: The grammar has been improved. Please see lines 221-222: “In the UK, a survey among people involved in PPIEP revealed a gender disparity in engagement, with women being more represented than men [18].”
