# Supplementary material for: Interest in public involvement, engagement and participation in research among patients with asthma: A secondary analysis of the EPI-ASTHMA study
Source: PLoS One. 2025 Sep 12;20(9):e0332220. doi: 10.1371/journal.pone.0332220 (PMC12431402; doi:10.1371/journal.pone.0332220)
Supplement: Attachment — Submitted filename: Response_to_Reviewers_auresp_4.docx [file pone.0332220.s005.docx]

Dear Prof. Manuela Mendonça Figueirêdo Coelho,
Academic Editor of PLOS One,

June 3rd, 2025

Title: Interest in Public Involvement, Engagement and Participation in Research among Patients with Asthma: A Secondary Analysis of the EPI-ASTHMA Study

Manuscript ID: PONE-D-25-04695

Dear Prof. Manuela Mendonça Figueirêdo Coelho,

Thank you for your consideration of our manuscript and for the useful comments provided.

I am looking forward to hearing from you.

Yours sincerely,

Cristina Jácome

**Point-by-point response**

Associate Editor:

1. Please, I would like to request the authors to carefully proofread the English.

Response: We have now carefully proofread the manuscript for English language and clarity. All changes made in response to your feedback are highlighted in blue within the revised document.
